# Supplementary material for: CX-5461 Treatment Leads to Cytosolic DNA-Mediated STING Activation in Ovarian Cancer
Source: Cancers (Basel). 2021 Oct 9;13(20):5056. doi: 10.3390/cancers13205056 (PMC8533980; doi:10.3390/cancers13205056)
Supplement: Supplementary file 1 [file cancers-13-05056-s001.zip › cancers-1383175-supplementary.pdf]

cancers MDPI

Supplementary materials: CX-5461 Treatment Leads to Cytosolic DNA-Mediated STING Activation in Ovarian Cancer

Robert Cornelison, Kuntal Biswas, Danielle C. Llaneza, Alexandra Harris, Nisha Sosale, Matthew J. Lazzara, and Charles N. Landen

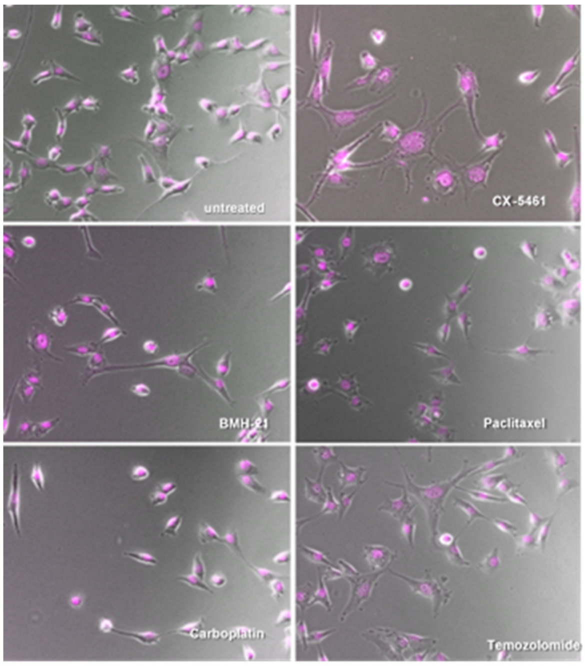

Figure S1. COV362 were treated with either vehicle or 1uM Cx-5461, 100nMBMH-21, 5nM paclitaxel, 50uM carboplatin, 100uM etoposide, and 250uM temozolomide for 24h, labeled with Sir-Hoechest. Representative images were shown.

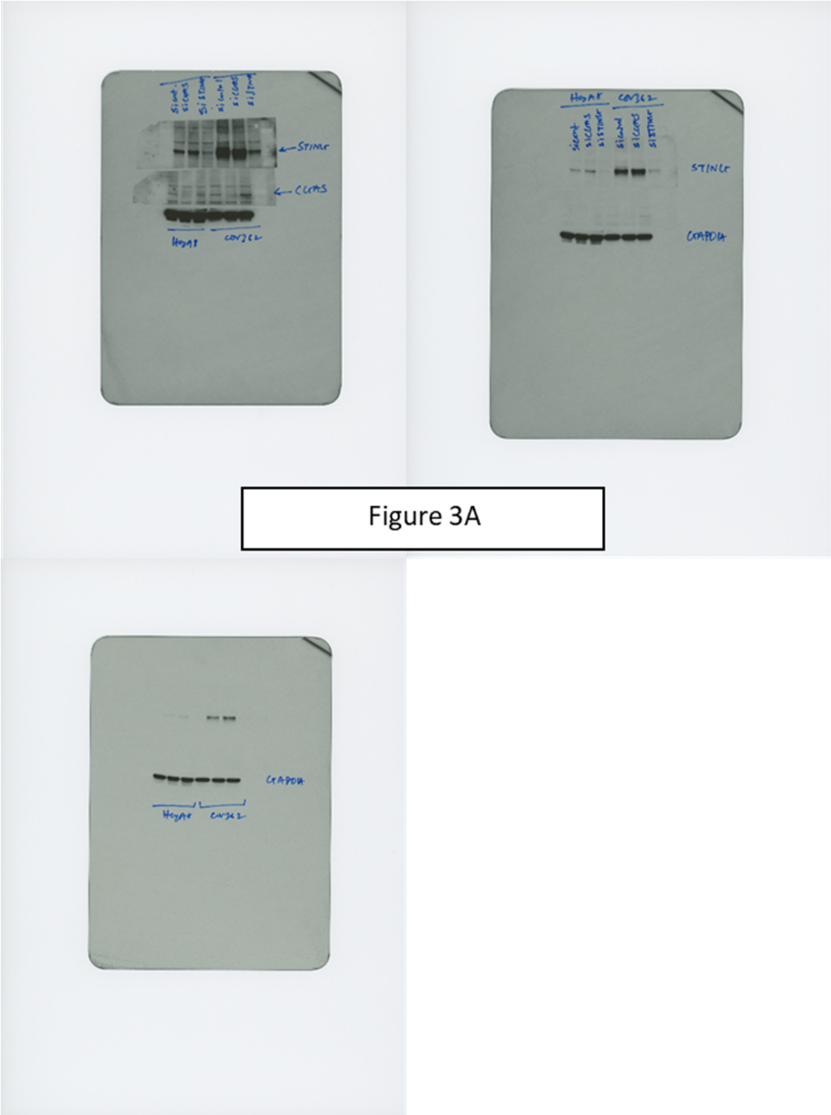

Figure 3A

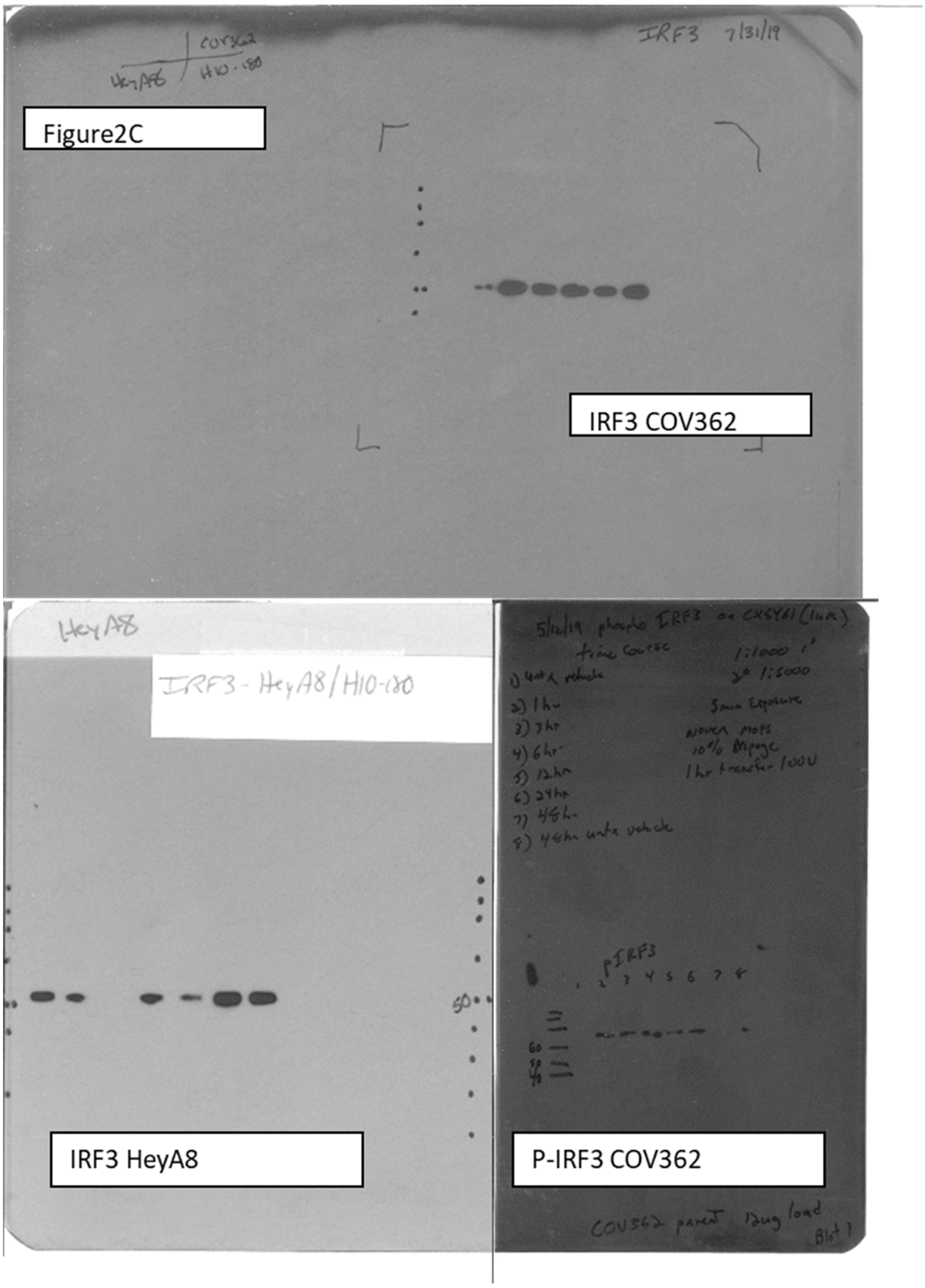

Figure2C
IRF3 7/31/19
IRF3 COV362
HeyA8
IRF3 - HeyA8/H10-180
IRF3 HeyA8
P-IRF3 COV362
pIRF3
COV362 parent

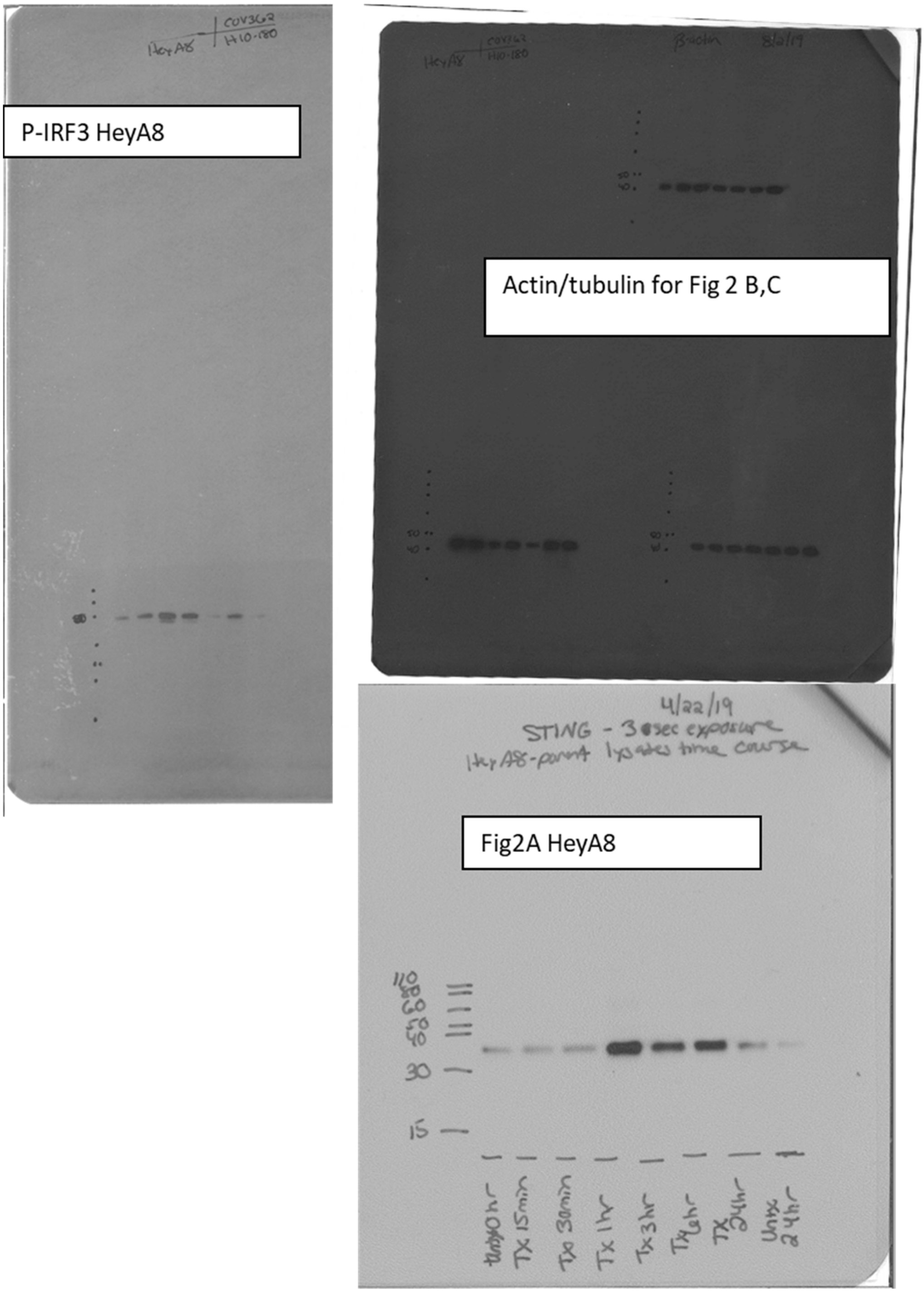

Figure S2. Original Western Blot images.

Supplemental Movie S1 link: https://app.box.com/s/bedhr1qntt42ml2u12kr4775ypqgys2g.

Supplemental Movie S2 link: https://app.box.com/s/bedhr1qntt42ml2u12kr4775ypqgys2g.
